# Elastin-derived peptide VGVAPG decreases differentiation of mouse embryo fibroblast (3T3-L1) cells into adipocytes

**DOI:** 10.1080/21623945.2020.1770525

**Published:** 2020-05-28

**Authors:** Konrad A. Szychowski, Bartosz Skóra, Jakub Tobiasz, Jan Gmiński

**Affiliations:** Department of Lifestyle Disorders and Regenerative Medicine, University of Information Technology and Management in Rzeszow, Rzeszow, Poland

**Keywords:** VGVAPG, elastin-derived peptides, 3T3, lipid accumulation

## Abstract

Elastin is a highly elastic protein present in connective tissue. As a result of protease activity, elastin hydrolysis occurs, and during this process, elastin-derived peptides (EDPs) are released. One of the constitutively repeating elastin and EDP building sequences is the hexapeptide VGVAPG. Therefore, the aim of our research was to define the effect of VGVAPG peptide on adipogenesis in a mouse 3T3-L1 cell line. 3T3-L1 cells were differentiated according to a previously described protocol and exposed to increasing concentrations of VGVAPG or VVGPGA peptide. The obtained results showed that VGVAPG peptide does not stimulate reactive oxygen species (ROS) production, caspase-1 activation, and 3T3-L1 cell proliferation. In the second part of the experiments, it was proved that VGVAPG peptide decreased lipid accumulation as measured by oil red O staining, which was confirmed by the profile of increased expression markers of undifferentiated preadipocytes. In our experiments, 10 nM VGVAPG added for differentiating to adipocytes increased the expression of Pref-1, serpin E1, and adiponectin as compared to rosiglitazone (PPARγ agonist)-treated group and simultaneously decreased the expression of VEGF and resistin as compared to the rosiglitazone-treated group. The obtained results show that VGVAPG peptide sustains 3T3 cells in undifferentiated state.

**Abbreviations**: DMSO: dimethyl sulphoxide; EBP: elastin-binding protein; EDPs: elastin-derived peptides; FBS: foetal bovine serum; *Glb1*: gene for beta-galactosidase; LDL: low-density-lipoprotein; PAI-1 (Serpin E1): plasminogen activator inhibitor-1; PBS: phosphate-buffered saline; PPARγ: peroxisome proliferator-activated receptor gamma; Pref-1: preadipocyte factor 1; ROS: reactive oxygen species; VEGF-A: vascular endothelial growth factor-A; VGVAPG: Val-Gly-Val-Ala-Pro-Gly; β-Gal: beta-galactosidase; ORO: oil red O; IBMX: 3-isobutyl-1-methylxanthine; H_2_DCFDA: 2ʹ,7ʹ-dichlorodihydrofluorescein diacetate; DMEM: Dulbecco’s Modified Eagle’s Medium; VVGPGA: Val-Val-Gly-Pro-Gly-Ala.

## 1. Introduction

Elastin is a highly elastic protein that occurs in connective tissue. Together with microfibrils, they are the main building blocks of elastin fibres. Skin, lung tissue, tendons, ligaments, and walls of blood vessels are rich in elastin [1]. This protein is synthesized in fibroblasts, chondroblasts, endothelial cells, and smooth muscle cells [2–5]. In physiological conditions, elastin-rich extracellular matrix (ECM) is slowly degraded; however, this process is accelerated during ageing or pathological conditions such as inflammation, atherosclerosis, and carcinogenesis in the breast, skin, and lung [6–9]. Elastin is degraded by proteolytic enzymes produced by monocytes, thrombocytes, neutrophils, lymphocytes, smooth muscle cells, skin fibroblasts, certain malignant tumour cells, and cells of adipose tissue [10–16]. As a result of protease activity, elastin hydrolysis occurs, and during this process, elastin-derived peptides (EDPs) are released [17]. One of the constitutively repeating elastin and EDP building sequences is the hexapeptide valine-glycine-valine-alanine-proline-glycine (VGVAPG) [18]. This peptide interacts with cells through a 67-kDa elastin-binding protein (EBP) located on the cell surface [18]. EBP is a catalytically inactive form of the alternatively spliced gene for β-galactosidase (*GLB1* gene) [19,20]. The second receptor for the VGVAPG peptide is galectin-3, which also has an important role in cell–ECM interactions [21]. Galectin-3 is mostly expressed in inflammatory cells [22,23]; however, its expression has been linked with tumour progression, and cancer aggressiveness [24–26]. There are several intracellular signalling pathways elicited by the EDP. The elastin receptor complex is linked to the G proteins which activation, opening of L-type calcium channels, and sequential activation of tyrosine kinases: FAK, c-Src, platelet-derived growth factor receptor kinase and then Ras-Raf- MEK1/2-ERK1/2 phosphorylation cascade [27].

It has been shown that EDPs and/or VGVAPG induce some biological effects such as cell proliferation, migration, differentiation, and inflammation through EBP activation [2,28–30]. Recently, it has been reported that VGVAPG peptide affects peroxisome proliferator-activated receptor gamma (PPARγ) mRNA and protein expression [31]. Furthermore, similar to EDPs, PPARγ is involved in cell proliferation, migration, and differentiation and most importantly in adipocyte differentiation [32]. Moreover, PPARγ is directly related to obesity in rodents, resulting in the overexpression of PPARγ in adipose tissue [33]. Adipocytes are characterized by secretion of many proteins such as adipocyte-derived vascular endothelial growth factor-A (VEGF-A) whose level is increased in obesity [34]; adiponectin related to insulin resistance [35]; preadipocyte factor 1 (Pref-1) that prevents fibroblasts from differentiation (adipose tissue generation) [36]; adipose-tissue secretory factor (ADSF/resistin) involved in low-density lipoprotein (LDL) level regulation [37]; and plasminogen activator inhibitor-1 (PAI-1/serpin E1) that plays a major role in insulin resistance [38]. All these proteins are also closely related to obesity, diabetes, atherosclerosis, or other conditions such as heart diseases. Hence, research studies are necessary to clarify the expression of these proteins in adipocytes. In addition to their role in glucose and lipid metabolism, adipocytes respond differentially to physiological indications or metabolic stress by releasing endocrine factors that regulate diverse processes, such as energy expenditure, appetite control, glucose homoeostasis, insulin sensitivity, inflammation and tissue repair [39]. Furthermore, reactive oxygen species (ROS) have been implicated as a contributor to both the onset and progression of insulin resistance and obesity, which are also associated with chronic low-grade inflammation [40]. To date, studies on the significance of VGVAPG peptide in the formation and metabolism of adipose tissue are limited. Blaise and team (2013) described that EDPs are involved in the development of insulin resistance in mice [41]. Moreover, Robert and colleagues, demonstrated that the concentration of anti-EDP antibodies of IgG is increased threefold in type 2 diabetic patients compared with the control population [42–44].

Therefore, the aim of our research was to determine the effect of VGVAPG peptide on adipogenesis in 3T3-L1 cell line that can function as an *in vitro* model for lipid accumulation.

## 2. Materials and methods

### 2.1. Reagents

Dulbecco’s Modified Eagle’s Medium (DMEM) without phenol red (10–013-CVR) and phosphate-buffered saline without calcium and magnesium (PBS) were purchased from Corning (Manassas, VA, USA). Trypsin, rosiglitazone, oil red O (ORO), Ac-YVAD-pNA (caspase-1 substrate), resazurin, penicillin, streptomycin, amphotericin B, 3-isobutyl-1-methylxanthine (IBMX), dexamethasone, insulin, RIPA buffer (product number R0278 – 50 mM Tris-HCl, pH 8.0, with 150 mM sodium chloride, 1.0% Igepal CA-630 (NP-40), 0.5% sodium deoxycholate, and 0.1% sodium dodecyl sulphate), 2ʹ,7ʹ-dichlorodihydrofluorescein diacetate (H_2_DCFDA), and dimethyl sulphoxide (DMSO) were purchased from Sigma-Aldrich (St. Louis, MO, USA). VGVAPG and VVGPGA peptides were synthesized by LipoPharm.pl (Gdańsk, Poland). Foetal bovine serum (FBS) was purchased from EURx (Gdańsk, Poland). Ki67 (EM1473) was purchased from Fine Biotech (Wuhan, China). Proteome profiler mouse adipokine array (ARY013) was purchased from R&D Systems, Inc. (Minneapolis, MN, USA). Stock solutions of VGVAPG and VVGPGA peptides were prepared in DMSO and then added to the DMEM medium. The final concentration of DMSO in the culture medium was always 0.1%.

### 2.2. 3T3-L1 cell culture, differentiation procedure, and treatment

Mouse embryonic fibroblast cell line 3T3-L1 was obtained from the American Type Culture Collection (ATCC, distributor: LGC Standards, Łomianki, Poland). The 3T3-L1 cell line was maintained in DMEM supplemented with 10% FBS, 100 U/mL penicillin, 0.10 mg/mL streptomycin, and 250 ng/mL amphotericin B. The cells were maintained at 37°C in a humidified atmosphere with 5% CO_2_. The cells were seeded in 96-well culture plates at a density of 6 × 10^3^ cells/well and in 6-well culture plates at a density of 3 × 10^5^ cells/well, and then initially cultured before the experiment for 24 h. Subsequently, the medium was replaced with a fresh one by increasing the concentrations (1, 10, 50, and 100 nM and 1, 10, 50, and 100 µM) of VGVAPG or VVGPGA, respectively. The levels of ROS, caspase-1 activation, resazurin reduction, and Ki67 protein expression were then measured.

Differentiation procedure was assessed according to Zebisch et al. (2012) with slight modifications [45] Briefly, 3T3-L1 cells were routinely cultured in basal medium (DMEM containing 10% FBS, 100 U/mL penicillin, 0.10 mg/mL streptomycin, and 250 ng/mL amphotericin B). The cells were seeded in 48-well plates at a density of 3 × 10^4^ cells/well (for ORO staining) or in 6-well plates at a density of 3 × 10^5^ cells/well (for protein). Forty-eight hours after seeding (day 2), cell differentiation was induced by changing the medium to basal medium containing 0.5 mM IBMX, 0.25 µM dexamethasone, and 1 µg/mL insulin. The experimental groups were negative control (without rosiglitazone), positive control with 2 µM rosiglitazone, and groups treated with 10 nM VGVAPG or 10 nM VVGPGA. After 48 h, the medium was changed to basal medium containing 1 µg/mL insulin for the next 48 h. After this time, the cell culture medium was again changed to basal medium, and the procedure was repeated after every two days. At 14 day (measured from cell seeding), ORO staining (48-well plates) and cell harvesting (6-well plates) were performed.

### 2.3. Production of ROS induced by VGVAPG peptide

The method for ROS determination is based on the oxidation of the fluorogenic dye 2ʹ,7ʹ-dichlorodihydrofluorescein diacetate (H_2_DCFDA), which is added to the cells before treatment with the studied peptide [46]. In the experiment, the cells were seeded in 96-well plates in DMEM medium supplemented with 10% FBS. After 24 h, the medium was replaced with DMEM without FBS containing the fluorogenic dye at 5 μM concentration for 45 min. The dye was then removed, and a series of dilutions of VGVAPG peptide (1 nM–100 µM) in DMEM supplemented with 1% FBS were added to the plate. To assess the ability of VGVAPG or VVGPGA to induce ROS production in 3T3-L1 cells, fluorescence was measured after 3, 6, 24, and 48 h. A microplate reader (FilterMax F5) was used to measure the maximum excitation and emission spectrum at 485 nm and 535 nm wavelengths, respectively.

### 2.4. Caspase-1 activity

Caspase-1 activity was assessed according to Nicholson et al. [47]. To measure caspase-1 activity, the cells were plated on 96-well plates and exposed to increasing concentrations of VGVAPG or VVGPGA peptides. Controls with or without DMSO vehicle were included in the experimental design to determine the effect of DMSO (results not shown). After thawing (−80ºC), the cells were lysed using lysis buffer (50 mM HEPES, pH 7.4, 100 mM NaCl, 0.1% CHAPS, 1 mM EDTA, 10% glycerol, and 10 mM DTT) at 10°C for 10 min. The lysates were incubated in the caspase-1 substrate Ac-YVAD-pNA at 37°C. After 30 min, the absorbance of the lysates at 405 nm was measured using a microplate reader (FilterMax F5). The amount of the colorimetric product was continuously monitored for 120 min.

### 2.5. Resazurin reduction assay

To determine changes in the level of cell metabolism/proliferation stimulated by VGVAPG, we used the redox dye resazurin. Depending on the metabolic activity of the cells, the dye shows both colorimetric and fluorometric changes. The metabolically active cells reduce nonfluorescent resazurin to fluorescent resorufin [48]. The cells were seeded on 96-well plates and exposed to increasing concentrations of VGVAPG or VVGPGA peptides at 24 h after seeding. After 48 or 72 h of incubation, the medium was removed and replaced with a new one (DMEM containing 1% FBS and 10% resazurin). The plates were then incubated for 60 min. Following this incubation, fluorescence was measured using a microplate reader (FilterMax F5) at the maximum excitation and emission spectrum of 530 nm and 590 nm wavelengths, respectively.

### 2.6. Enzyme-linked Immunosorbent Assay (ELISA) for Ki67

The level of Ki67 protein was determined after 48 h of exposure to 1, 10, 50, and 100 nM and 1, 10, 50, and 100 µM of VGVAPG or VVGPGA treatment, respectively, by enzyme-linked immunosorbent assay (ELISA). These proteins were specifically detected by ELISA and subsequently subjected to quantitative sandwich enzyme immunoassay. The assay was performed according to the manufacturer’s instructions. Briefly, a 96-well plate was precoated with monoclonal antibodies specific to Ki67. Standards and the collected cell extracts were added to the wells and incubated for 90 min at 37°C. Next, after removing the liquid, 100 µL of biotinylated detection antibodies were added for 60 min. After 3 times washing to remove any unbound substances, horseradish peroxidase-conjugated avidin was added. Following additional washing, 90 µL of substrate solution was added to the wells for 15 min. Then, 50 µL of the reaction termination solution was added, and the absorbance was measured at 450 nm. The obtained values were proportional to the amount of Ki67. The total protein concentration was determined in triplicate in each sample by using a Thermo Fisher NanoDrop device.

### 2.7. Oil Red O (ORO) staining and quantification

Oil red O (ORO) staining was performed according to Zebish et al. with slight modifications [45]. To perform the staining of cells, 1 day before the experiment, a stock solution of ORO was prepared by dissolving 1 g of ORO in 100 mL of absolute isopropanol. The prepared solution was left standing overnight on a stirrer at room temperature. The solution was then filtered through a filter paper. The working solution was prepared just before staining by mixing the stock solution with distilled water (3:2). The cells were washed twice with PBS and incubated for 1 h at room temperature in a 10% formalin solution. After incubation, the cells were washed twice with distilled water and once with 60% isopropanol. The cells were then incubated with the working solution of the ORO dye for 15 min. The cells were washed 5 times with distilled water, and after the 5th wash, the cells were photographed. The cells were then washed three times with 60% isopropanol. Finally, the cells were washed with 100% isopropanol. The samples were then transferred to new 96-well plates to measure absorbance at 450 nm. The blank was 100% isopropanol.

### 2.8. Proteome profiler mouse adipokine array

Briefly, cells were collected in RIPA buffer, and protein concentration was determined using a Thermo Fisher NanoDrop device. A protein profiler membrane was prepared according to the manufacturer’s protocol. Next, equal amounts of protein (500 µg/mL) from the experimental group (Control – undifferentiated; Rosiglitazone – positive control, cell differentiated with 2 µM rosiglitazone; VVGPGA – cell differentiated with 10 nM VVGPGA, VGVAPG – cell differentiated with 10 nM VGVAPG) were mixed with 15 μL of reconstituted mouse adipokine detection antibody cocktail and incubated at room temperature for 1 h. The sample/antibody mixtures were added to 4-well plates and incubated overnight at 2–8°C on a rocking platform shaker. On the next day, the membranes were washed in a wash buffer two times for 10 min. After this step, the membranes were incubated with streptavidin-HRP antibody for 30 min at room temperature on a rocking platform shaker. After washing for three times, the chemiluminescent reagent was added to each membrane and incubated for 1 min. In the last step, the membranes were placed in a C-DiGit Blot Scanner (LI-COR) and scanned for chemiluminescence. Quantification of protein band densitometry was carried out using ImageJ 1.52a software.

### 2.9. Statistical analysis

Data are presented as mean ± SD of three independent experiments. Each treatment was repeated six times (n = 6) and measured in triplicate. The data were normalized to the vehicle-treated control cells and presented as percentage of control. The data were analysed by one-way analysis of variance (ANOVA) followed by Tukey’s multiple comparison procedure ***p < 0.001, **p < 0.01, and *p < 0.05 vs. the control were considered statistically significant.

## 3. Results

### 3.1. Measurement of ROS production

**Figure 1.**
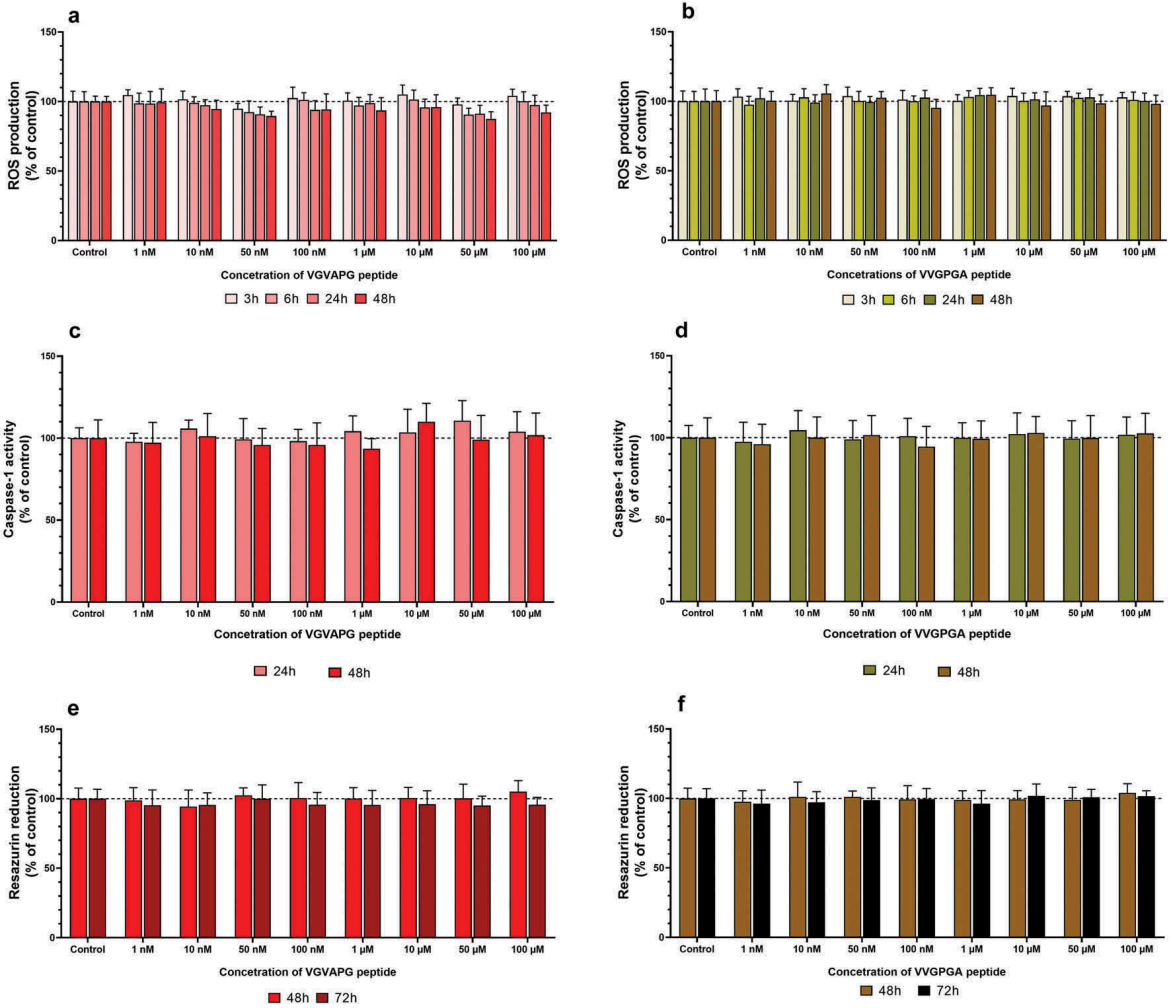
Effect of the increasing concentrations of VGVAPG or VVGPGA peptides on ROS production (a and b), activity of caspase-1 (c and d), and resazurin reduction (e and f). Measurement were performed after 3, 6, 24, and 48 h for ROS; after 24 and 48 h for caspase-1 activity; and after 48 and 72 h for resazurin reduction assay in mouse 3T3-L1 cell line. Data are expressed as mean ± SD of three independent experiments, each of which comprised six replicates per treatment group

After 3T3-L1 cell treatment with increasing concentrations of VGVAPG or VVGPGA peptides, no changes were found in ROS production in all studied time intervals (3, 6, 24, and 48 h) (Figure 1(a,b).

### 3.2. Measurement of caspase-1 activity

After 3T3-L1 cell treatment with increasing concentrations of VGVAPG or VVGPGA peptides, no significant changes were found in caspase-1 activity in both 24 and 48 h time intervals (Figure 1(c,d)).

### 3.3. Measurement of cell metabolism

After 3T3-L1 cell treatment with increasing concentrations of VGVAPG or VVGPGA peptides, no significant changes were found in the resazurin reduction assay in both 48 and 72 h time intervals (Figure 1(e,f)).

### 3.4. Measurement of Ki67 protein level

**Figure 2.**
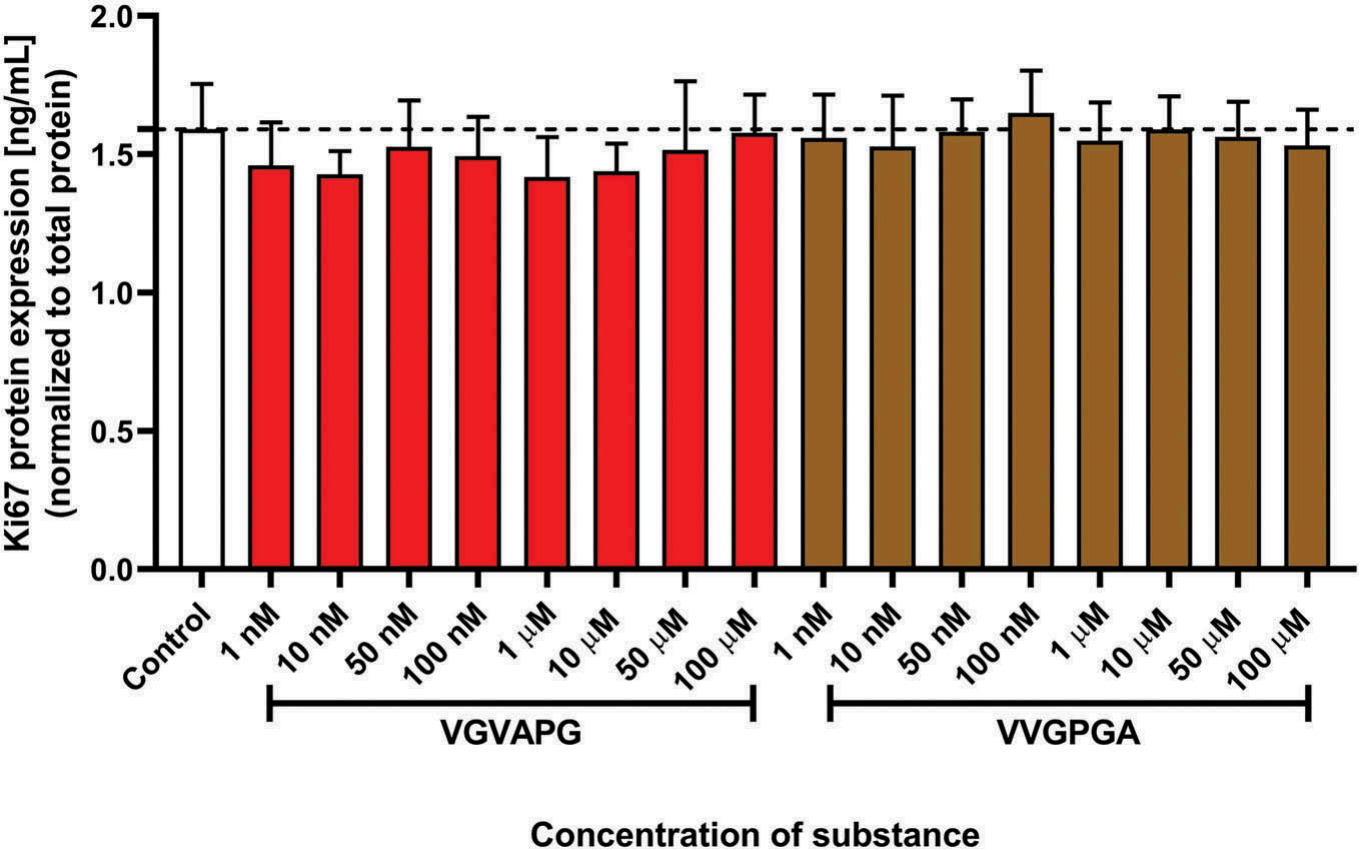
Effect of the increasing concentrations of VGVAPG or VVGPGA peptides on the expression of Ki67 protein. Ki67 level was measured by the ELISA method after 48 h of exposure of 3T3-L1 cell line to the studied peptides. Data are expressed as mean ± SD of three independent experiments, each of which comprised six replicates per treatment group

After 48 h of 3T3-L1 cell exposure to increasing concentrations of VGVAPG or VVGPGA peptide, no significant changes were found in Ki67 protein expression level (Figure 2).

### 3.5. Measurement of lipid accumulation by ORO staining

**Figure 3.**
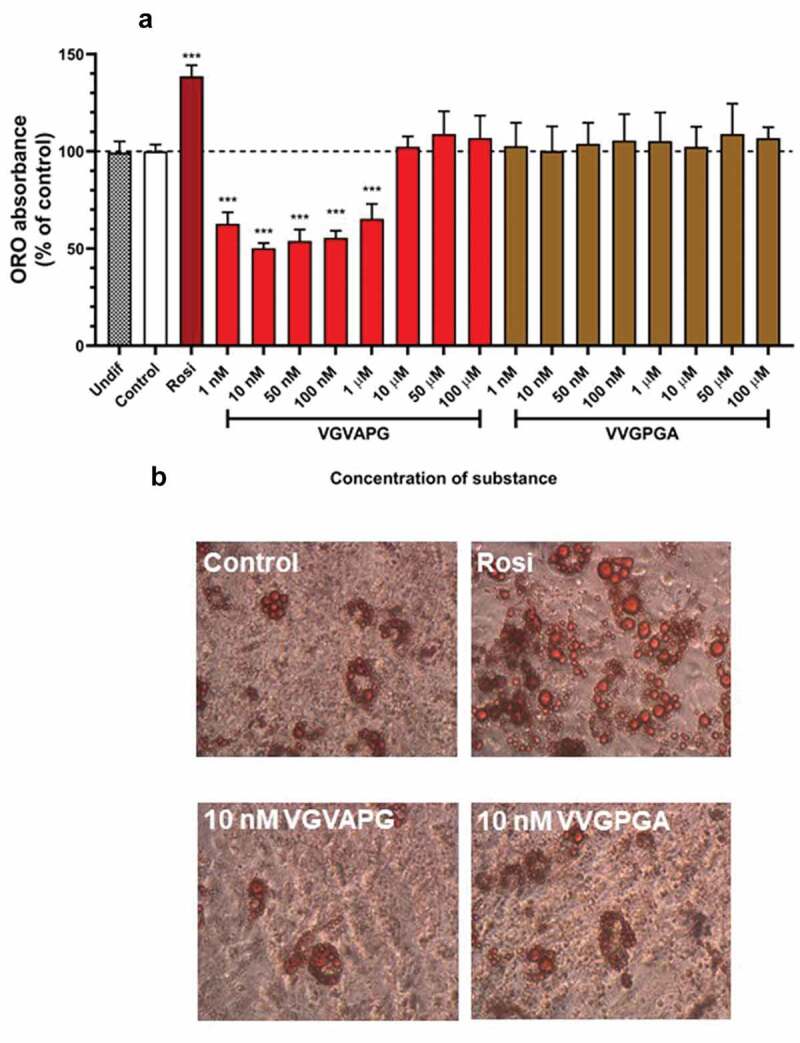
Effect of the increasing concentrations of VGVAPG or VVGPGA (a) peptides on lipid accumulation in 3T3-L1 cell line. Undif – undifferentiated cells; Control – cells differentiated with IBMX, dexamethasone, insulin, without rosiglitazone; Rosi – positive control, differentiated cells with IBMX, dexamethasone, insulin and 2 µM rosiglitazone; groups treated with VGVAPG or VVGPGA were differentiated with IBMX, dexamethasone, insulin and appropriate concentration of VGVAPG or VVGPGA. ORO staining (b) and quantification were performed after 14 days of differentiation

After 14 days of differentiation using IBMX, dexamethasone, insulin, and other appropriate compounds (increasing concentrations of VGVAPG or VVGPGA or 2 µM rosiglitazone), ORO staining and its quantification were performed. Undifferentiated 3T3-L1 cells (Undif); cells differentiated using only IBMX, dexamethasone, and insulin (Control); and cells differentiated using IBMX, dexamethasone, and insulin and increasing concentrations of VVGPGA or VGVAPG (1, 10, 50, and 100 nM and 1, 10, 50, 100 µM, respectively) did not accumulate lipid (Figure 3). In cells differentiated using IBMX, dexamethasone, insulin, and 2 µM rosiglitazone (Positive Control), lipid accumulation significantly increased as compared to that in Control without rosiglitazone (increase by 38.57%). In differentiated 3T3-L1 cells with VGVAPG concentrations in range of 1–100 nM and 1 µM peptide, lipid accumulation decreased as compared to that in Control (decrease from 50.01% to 34.54%). However, VGVAPG peptide treatment in the range of 10–100 µM did not affect lipid accumulation as compared to that in Control (Figure 3).

### 3.6. Proteome profiler mouse adipokine array

**Figure 4.**
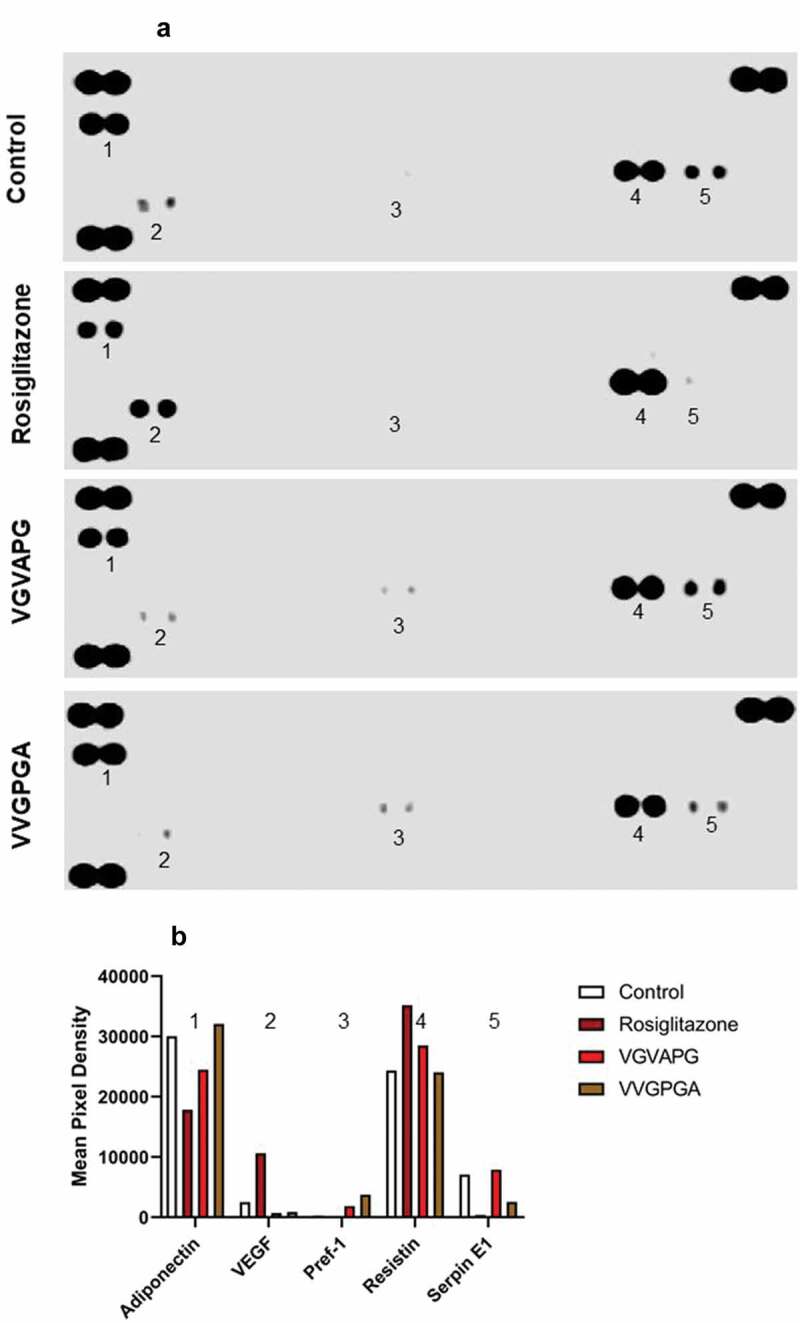
Effect of 10 nM VGVAPG or VVGPGA peptides on the protein expression profile in 3T3-L1 cell line. Control – undifferentiated cells; Rosiglitazone – positive control, cells differentiated with IBMX, dexamethasone, insulin, and 2 µM rosiglitazone; VGVAPG – cells differentiated with IBMX, dexamethasone, insulin, and 10 nM VGVAPG; VVGPGA – negative control VVGPGA peptide that does not activate EBP, cells differentiated with IBMX, dexamethasone, insulin, and 10 nM VVGPGA (a). Protein measurement was performed after 14 days of differentiation. Densitometry was performed by ImageJ 1.52a software (b)

After 14 days of differentiation using IBMX, dexamethasone, insulin, and other appropriate compounds (10 nM VGVAPG, 10 nM VVGPGA, or 2 µM rosiglitazone), protein quantification with the proteome profiler array was performed. Two controls (Control: undifferentiated cells and VVGPGA Control: cells differentiated with 10 nM VVGPGA – that peptide that does not activate EBP) were used. Cells differentiated with rosiglitazone were used as a positive control (Figure 4).

VGVAPG peptide decreased the expression of adiponectin as compared to that in Control and VVGPGA cells. However, rosiglitazone caused a higher reduction of adiponectin expression than VGVAPG peptide.

Further, VGVAPG peptide decreased the expression of VEGF compared to that in Control and VVGPGA cells, but rosiglitazone significantly increased VEGF expression in 3T3-L1 cells.

In cells treated with IBMX, dexamethasone, insulin, and VGVAPG or VVGPGA, the expression of Pref-1 was increased significantly as compared to that in undifferentiated cells. However, Pref-1 expression was lower in cells treated with VGVAPG than in cells treated with VVGPGA. Pref-1 expression was not detected in the group treated with rosiglitazone.

The expression level of resistin did not differ in Control and VVGPGA cells. Cells treated with VGVAPG peptide showed increased level of resistin expression. Resistin expression was much higher in cells treated with rosiglitazone than in the VGVAPG peptide-treated group.

In cells treated with VGVAPG peptide, the expression of serpin E1 was similar to that of undifferentiated cells and much higher than that in cells treated with VVGPGA or rosiglitazone. The lowest expression of serpin E1 was noted in the rosiglitazone-treated group (Figure 4).

## 4. Discussion

It is well known that EDPs or VGVAPG increase ROS production in different cells such as murine monocytes and astrocytes, human fibroblasts, and neuroblastoma (SH-SY5Y) cells [49–53]. Moreover, ROS are key signalling molecules that play an important role in the progression of inflammatory disorders. Interleukin-1 beta (IL-1β) is one of the key pro-inflammatory cytokines in an organism [54]. IL-1β is formed when its inactive precursor pro-IL-1β is activated by limited proteolysis through the interleukin-1-beta-converting enzyme (ICE), which is currently known as caspase-1 [55]. ROS activation can initiate or result in an inflammation process in which caspase-1 plays an important role [56]. Caspase-1 is a member of the intracellular cysteine protease family that mediates inflammation and activates IL-1β and IL-18 [57]. Mice lacking IL-18 become obese and insulin resistant, and both IL-1β and IL-18 play a role in overall energy balance [58]. Moreover, it has been reported that in mouse with caspase-1 knocked out, obesity develops similar to mice with IL-18 deficiency [59]. Interestingly, the IL-18 protein level is upregulated in the adipose tissue of obese mice [60]. Furthermore, ROS production and/or inflammation process can initiate cell death and decrease cell metabolism, but in some cases, a contrasting effect might be observed and metabolism and/or cell proliferation could increase [61,62]. Our data show that VGVAPG peptide did not increase ROS production and induce caspase-1 activation and cell proliferation in all studied concentrations and time intervals in 3T3-L1 cell line. To date, previous studies have shown that EDPs and/or VGVAPG peptide increase the production and/or secretion inflammatory markers such as IL-1α, IL-1β, and IL-6 in *ligamentum flavum* cells, synovial cells, and melanoma cell lines [63–65]. On the other hand, our previous studies show that VGVAPG peptide increases the expression of PPARγ and decreases the expression of nuclear factor kappa-light-chain-enhancer of activated B cells (NF-κB) and production of IL-1β in mouse astrocytes in a PPARγ-dependent manner, which suggest that the effect of VGVAPG is tissue dependent [31,66]. Interestingly, our latest study revealed that caspase-1 activity increases, which suggests that this caspase does not play an inflammatory role in mouse astrocytes [66].

ORO staining and its quantification are a recognized marker for determining the maturity of adipocytes [67]. This method is based on a correlation between higher values of ORO dye in the fully matured adipocytes than in less differentiated or undifferentiated cells. This method has been successfully validated, indicating that the ORO assay can be used to analyse adipocyte differentiation [68–70]. Our obtained data show that VVGPGA peptide did not induce lipid accumulation in 3T3-L1 cells, while VGVAPG peptide in the range of 1 nM to 1 µM decreased lipid accumulation as compared to that in control. Based on the obtained data from ORO measurement for further research, we chose 10 nM VGVAPG peptide as the concentration to evaluate the level of activated proteins during the differentiation process in adipocytes.

During the differentiation process in preadipocytes, the expression profile of proteins changed in 3T3-L1 cell line. Our data show that VGVAPG peptide altered the expression profile of Pref-1, serpin E1, adiponectin, VEGF, and resistin which are the key proteins involved in developing obesity and/or insulin resistance. In our experimental model, VGVAPG peptide increased the expression of Pref-1, serpin E1, and adiponectin as compared to rosiglitazone, which is a PPARγ agonist used to trigger differentiation of 3T3-L1 cells into mature adipocytes. It is well known that Pref-1 is highly expressed in 3T3-L1 cells but shows reduced expression during adipocyte differentiation [71]. Therefore, Pref-1 serves as an excellent marker for preadipocytes. Pref-1 is also an inhibitor of adipogenesis, and its constitutive expression inhibits 3T3-L1 adipocyte differentiation [36]. Moreover, in our experiments VGVAPG peptide increased PAI-1 (synonym serpin E1) expression, which is linked to obesity and insulin resistance [72]. Liang et al. (2006) reported that the overexpression of PAI-1 by adenovirus-mediated gene transfer in 3T3-L1 adipocytes inhibited differentiation and reduced PPARγ expression [72]. Finally, compared to the rosiglitazone-treated group, VGVAPG peptide increased adiponectin expression in 3T3-L1 cells. Adiponectin produced by adipocytes is an important vascular protective adipocytokine that possesses antidiabetic, antiatherogenic, and anti-inflammatory properties [73]. Furthermore, adiponectin is an important insulin-sensitizing adipocytokine that is downregulated in insulin resistance and obesity, and replenishment of this protein in adiponectin-deficient state improves insulin sensitivity [74]. Additionally, leptin, adiponectin, and glucose transporter type 4 (Glut 4) are the major target genes in adipose tissue for PPARγ, and therefore, they are essential for maintaining homoeostasis of carbohydrate metabolism. It was shown that any disorders related to the regulation of these genes may contribute to the development of obesity due to the important role of these genes in hunger regulation (leptin), glucose transport (Glut 4), and insulin susceptibility (adiponectin) [75–79].

In our experimental model, VGVAPG peptide decreased the expression of VEGF and resistin compared to that observed in the rosiglitazone-treated group. Vascular endothelial growth factor (VEGF) is the family of secreted polypeptides, characterized as cysteine-knot superfamily of hormones, including VEGF-A and others, which are formed by alternative splicing process [80]. VEGF is recognized as a key factor in normal and abnormal angiogenesis and regulates multiple biological responses in endothelial cells, including cell proliferation, migration, survival, and production of vasoactive mediators [81]. For example, VEGF-A released from adipocytes promotes angiogenesis and thereby ameliorates the local hypoxia-induced adipose inflammation and insulin resistance [82]. Our data suggest that reducing VEGF expression by VGVAPG could reduce angiogenesis in adipose tissue.

The last studied protein resistin promotes 3T3-L1 preadipocyte differentiation and is an important mediator of obesity-induced insulin resistance; moreover, this protein could be secreted by adipocytes and is involved in insulin regulation [83]. According to literature data, the level of resistin increases in 3T3-L1 during the differentiation process [84]. On the other hand, in adult/mature adipocytes, resistin levels were shown to be decreased by rosiglitazone, and ligand-induced PPARγ activation strongly downregulated resistin expression; thus, this protein has been proposed as a therapeutic target of PPARγ ligands, which are used clinically to improve insulin sensitivity [85–87]. In our study, the VGVAPG peptide decreased the expression of resistin as compared to that in rosiglitazone-treated cells. Because resistin is strictly controlled by PPARγ activation, our data allow to assume that VGVAPG peptide by interaction with this receptor can control resistin gene expression [88]. Interaction between PPARγ and VGVAPG has been proven in our previous study, in which VGVAPG peptide was shown to affect PPARγ expression and PPARγ-mediated effects in mouse astrocytes and SH-SY5Y cells [31,49]. As mentioned above, PPARγ is involved in resistin expression, which is strictly related to obesity and type 2 diabetes. To date, Blaise and co-workers (2013) described that EDPs are involved in the development of insulin resistance in mice [41]. Moreover, several papers, demonstrated that the concentration of anti-EDP antibodies of IgG is increased three fold in type 2 diabetic patients compared with the control population [42–44]. Based on that we believed that, VGVAPG peptide affecting the PPARγ pathway could first prevent the maturation of preadipocytes and next make it difficult to break insulin resistance. According to available literature data which described mechanisms of action EDPs and PPARγ we hypothesized that in our experimental model VGVAPG could interfere with MAP-kinase [27,89]. MAP-kinase is commonly known as an agent taking part in many cell types differentiation processes, such as: adipocytes [90], osteocytes [91] or neurons [92], therefore we think that VGVAPG can affect differentiation process of 3T3-L1 cells in PPARγ-MAPK-dependent way.

Taking into account all the above data, our obtained results show that VGVAPG peptide sustains 3T3 cells in less undifferentiated state. This could potentially have many repercussions such as preventing maturation of preadipocytes, possibility of carcinogenesis in undifferentiated cells, and making it difficult to break insulin resistance.

## 5. Conclusions

**Figure 5.**
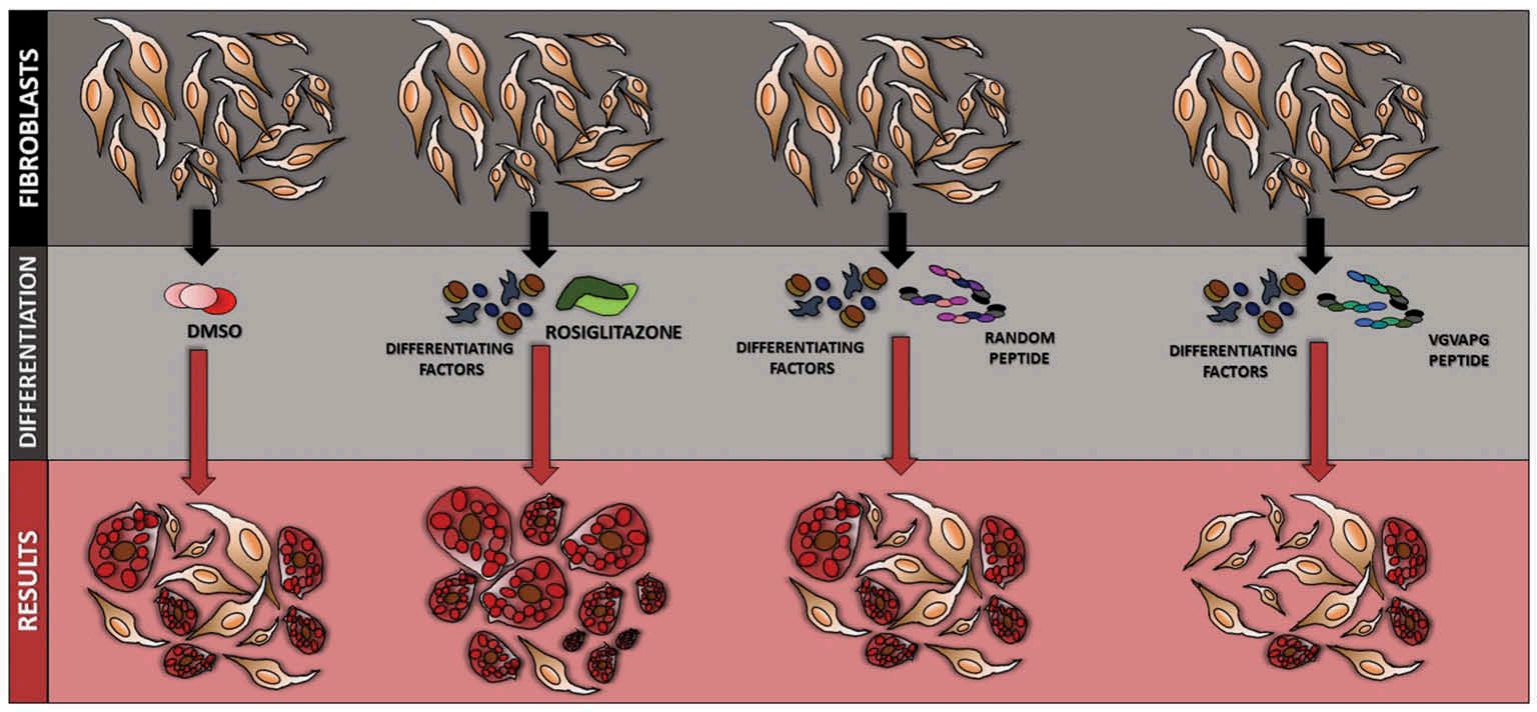
Diagram showing the effect of VGVAPG peptide on the differentiation process in 3T3 cells

The present study is the first to describe that VGVAPG peptide does not increase ROS production and induce caspase-1 activation and cell proliferation in all studied time intervals in 3T3-L1 cell line. Moreover, VGVAPG peptide decreased lipid accumulation measured by ORO assay and increased the expression of Pref-1, serpin E1 and adiponectin as compared to that in rosiglitazone (PPARγ agonist used to trigger differentiation of 3T3-L1 cells)-treated group, while simultaneously decreasing the expression of VEGF and resistin. Our obtained results show that VGVAPG peptide sustains 3T3 cells in less undifferentiated state (Figure 5). However, because of the lack of sufficient data explaining the molecular mechanism of action of VGVAPG peptide in adipose tissue, more studies are necessary on this topic.

## Highlights

- VGVAPG peptide does not stimulate ROS production and activation of caspase-1 in 3T3-L1 cells
- VGVAPG peptide does not induce proliferation of 3T3-L1 cells
- VGVAPG peptide decreases differentiation of 3T3-L1 cells into adipocytes
- VGVAPG peptide decreases lipid accumulation as measured by ORO assay
- VGVAPG peptide increases expression of Pref-1, serpin E1 and adiponectin as compared to rosiglitazone
- VGVAPG peptide decreases expression of VEGF and resistin as compared to rosiglitazone

## Author statement

Konrad A. Szychowski: Conceptualization, Methodology, Validation, Formal analysis, Investigation, Writing - Review & Editing, Project administration, Data Curation, Writing - Original Draft. Bartosz Skóra.: Data curation, Writing- Original draft preparation. Jakub Tobiasz: Investigation. Jan Gmiński: Supervision Writing - Review & Editing, Funding acquisition.
